# KLF4 and CD55 expression and function depend on each other

**DOI:** 10.3389/fimmu.2023.1290684

**Published:** 2024-02-09

**Authors:** Feng-Qi An, Guangjin Zhou, Micah T. Harland, Wasim Hussain, Michael G. Strainic, Mukesh K. Jain, M. Edward Medof

**Affiliations:** ^1^ Institute of Pathology Case Western Reserve University and Cardiovascular Research Institute, Department of Medicine, Harrington Heart and Vascular Institute, University Hospitals Cleveland Medical Center, Cleveland, OH, United States; ^2^ Cardiovascular Research of Medicine, Case Western Reserve University, Cleveland, OH, United States

**Keywords:** CD55, KLF4, CREB, CBP, CD55 (DAF), Kruppel like factor 4(KLF4) gene, decay accelerating factor (CD55), CREB binding protein (CBP)

## Abstract

The transcription factor Kruppel-like factor 4 (KLF4) regulates the expression of immunosuppressive and anti-thrombotic proteins. Despite its importance in maintaining homeostasis, the signals that control its expression and the mechanism of its transactivation remain unclarified. CD55 [aka decay accelerating factor (DAF)], now known to be a regulator of T and B cell responses, biases between pro- and anti-inflammatory processes by controlling autocrine C3a and C5a receptor (C3ar1/C5ar1) signaling in cells. The similarity in CD55’s and KLF4’s regulatory effects prompted analyses of their functional relationship. In vascular endothelial cells (ECs), CD55 upregulation accompanied KLF4 expression via a p-CREB and CREB Binding Protein (CBP) mechanism. In both ECs and macrophages, CD55 expression was essential for KLF4’s downregulation of pro-inflammatory/pro-coagulant proteins and upregulation of homeostatic proteins. Mechanistic studies showed that upregulation of KLF4 upregulated CD55. The upregulated CD55 in turn enabled the recruitment of p-CREB and CBP to KLF4 needed for its transcription. Activation of adenylyl cyclase resulting from repression of autocrine C3ar1/C5ar1 signaling by upregulated CD55 concurrently led to p-CREB and CBP recruitment to KLF4-regulated genes, thereby conferring KLF4’s transactivation. Accordingly, silencing CD55 in statin-treated HUVEC disabled CBP transfer from the E-selectin to the eNOS promoter. Importantly, silencing CD55 downregulated KLF4’s expression. It did the same in untreated HUVEC transitioning from KLF4^low^ growth to KLF4^hi^ contact inhibition. KLF4’s and CD55’s function in ECs and macrophages thus are linked via a novel mechanism of gene transactivation. Because the two proteins are co-expressed in many cell types, CD55’s activity may be broadly tied to KLF4’s immunosuppressive and antithrombotic activities.

## Introduction

Kruppel-like factor 4 (KLF4) is a transcription factor that induces the expression of anti-inflammatory and anti-thrombotic proteins (1, 2). Decreases in its function are implicated in proinflammatory disorders including atherosclerosis, autoimmunity and cancer (3, 4) and increases in homeostatic processes, including vascular tone, mitochondrial homeostasis and tissue regeneration (5–8). Its overexpression both in human and mouse endothelial cells (ECs) induces cardioprotective eNOS and thrombomodulin (TM), whereas its knockdown leads to enhancement of TNF-α-and IL-1β-induced vascular cell adhesion molecule-1 (VCAM-1) and tissue factor expression (9). Myeloid deficiency of KLF4 confers a pro-inflammatory phenotype resulting in obesity and insulin resistance (10). Consistent with this, KLF4 expression in adipose tissue macrophages is reduced in obese diabetic subjects (10).

CD55 [aka decay accelerating factor (DAF)] is a cell-associated complement regulatory protein. It originally was characterized as a shield that protects self-cells from attack by plasma complement proteins (11, 12). It is now known however to control many functions of cells by virtue of regulating complement that is endogenously produced by the cells in which it is expressed (13) in distinction to (liver produced) complement in plasma. This insight initially came from studies of immune cell activation (14) which showed that interacting dendritic cells (DCs) and CD4^+^ T cells generate C3a and C5a activation fragments from endogenously synthesized C3 and C5 proteins and that these anaphylatoxins establish autocrine signaling loops with C3a receptor (C3ar1) and C5a receptor (C5ar1) G protein coupled receptors (GPCRs) in each partner. CD55 regulates the intensity of this autocrine GPCR signaling. Downregulated CD55 potentiates C3ar1/C5ar1 signaling and drives T effector-cell (Teff) responses (14). Conversely, upregulated CD55 represses C3ar1/C5ar1 signaling and imprints Foxp3^+^ T regulatory cell (Treg) commitment (15). In CD4^+^ T cells, CD55 thus biases between pro-and anti-inflammatory immune responses. The cellular signaling underlying these opposing cellular outcomes is that potentiated C3ar1/C5ar1 signaling occurring with downregulated CD55 represses adenylyl cyclase and activates phosphoinositide-3 kinase γ (PI-3Kɣ) (14), whereas suppressed C3ar1/C5ar1 occurring with upregulated CD55 has the opposite effects. Important for this study, lifted restraint on adenylyl cyclase occurring with upregulated CD55 (15) enables activation of protein kinase A (PKA) needed for the generation of p-CREB.

Recent work deriving from the above findings showed that in oral feeding (classical ovalbumin model) and transplantation (16, 17), CD55’s regulation of autocrine C3ar1/C5ar1 signaling in DCs and macrophages biases between inflammatory *vs* tolerogenic processes. In that context (16), CD55's upregulation represses Toll Like Receptor (TLR) signaling and promotes M2 anti-inflammatory polarization while concurrently suppressing the M1 proinflammatory phenotype. In B cell activation (18), upregulated CD55 biases in favor of repressed as opposed to heightened antibody (Ab) production, and simultaneously diminishes affinity maturation (AFM) of the Ab and its class switch recombination (CSR) to effector Ig subtypes. In the context of cell growth (19), CD55’s regulation of C3ar1/C5ar1 signaling in vascular endothelial cells (ECs), smooth muscle cells (SMCs) (20), and myocytes (20) controls vascular endothelial cell growth factor (VEGF-A), platelet derived growth factor (PDGF), and epidermal growth factor (EGF) viability and mitotic signaling in the respective cell types. These newly uncovered insights together with the finding that CD55 control of C3ar1/C5ar1 signaling operates in multiple cell types to control cellular functions (16, 19, 20) have connected CD55’s upregulation with homeostatic processes physiologically (14, 19) and its downregulation with proinflammatory processes pathologically (14, 21). These broad effects of CD55 indicate that regulation of cellular processes may be its primary function.

Prompted by CD55’s homeostatic effects, we tested whether its regulation of autocrine C3ar1/C5ar1 signaling is involved in KLF4’s anti-inflammatory and anti-thrombotic functions. A report by others found that KLF4 upregulates CD55 expression on intestinal enterocytes (IECs) (22). In accordance with that finding in IECs, our studies linked KLF4 expression with CD55 expression in ECs, macrophages and other vascular and immune relevant cell types. The parallel functions of CD55 with KLF4 prompted studies of the effects of CD55 on KLF4 (in distinction to those of KLF4 on CD55).

The experiments herein show that many immunosuppressive and antithrombotic effects in ECs, macrophages, and immune cells ascribed to KLF4 are tied to CD55’s repression of autocrine C3ar1/C5ar1 signaling. The data thus provide mechanistic insight into how KLF4 expression is regulated and how it can transactivate to multiple KLF4 target genes in the same cell.

## Methods

### Cell lines

Human Umbilical Vein Endothelial Cells (HUVEC) purchased from Cambrex Corp. were cultured in complete EGM-2 medium (Lonza, Switzerland). Passage 3-10 HUVEC maintained in confluency for up to 3 days were used. K562 cells were grown in RPMI 1640 media containing 10% fetal bovine serum (FBS) and pen/strep. HeLa cells and Raw 264.7 cells were cultured in DMEM media containing 10% FBS and pen/strep. Anti-CBP mAb that was used in the ChIP assays recognizes both CBP and p300 was from ABCAM (Cambridge UK).

### qPCR and semi quantitative PCR

Total RNA was extracted using the QIAGEN mini-RNeasy kit. Six to nine hundred ng of RNA was reverse transcribed by oligo dT primers with Superscript III (Invitrogen). Real-time PCR was performed on an Applied Biosystems Prism 7500 apparatus. The primers used for amplification were as follows: Human GAPDH forward 5’- ACCCACTCCTCCACCTTTGA -3’ reverse 5’- CTGTTGCTGTAGCCAAATTCGT -3’; human CD55: forward 5’- TGAAACAACCCCAAATAAAGGAA -3’ reverse 5’- CTAGCGTCCCAAGCAAACCT -3’; human KLF4: forward 5’- CCCAATTACCCATCCTTCCT -3’ reverse 5’-ACGATCGTCTTCCCCTCTTT- 3’; mouse actin: forward 5’- AGAGGGAAATCGTGCGTGAC -3’ reverse 5’- CAATAGTGATGACCTGGCCGT -3’; mouse CD55: forward 5’- ACTGTTGATTGGGACGATGAG -3’ reverse 5’- TGGTGGCTCTGGACAATGTA -3’; mouse KLF4: forward 5’- CGGGAAGGGAGAAGACACT -3’ reverse 5’- GAGTTCCTCACGCCAACG -3’. Mean threshold cycle differences in the cDNAs were normalized against those for GAPDH or actin (internal control) in the corresponding preparation.

### Affymetrix gene array analysis

Total RNA was prepared by using QIAGEN mini-RNeasy kit. Gene expression profiling was performed using Affymetrix oligonucleotide-based Human Genome U133 plus 2.0 chips. All procedures and data analyses were performed as previously described (23). Gene arrays were performed by the Case Comprehensive Cancer Center Gene Expression core facility. The arrays consisted of 47,000 probe sets with expressed sequence tags (corresponding to ~14000 genes).

### KLF4 expression plasmids and adenoviral constructs

KLF4's cDNA sequence was obtained from the human GenBank accession # NM-004235. The KLF4 gene was inserted into pEGFP-N1 vector (Clontech) upstream of green fluorescent protein (GFP) between Hind III and Kpn I sites or into pTracer-EF/V5 His-C vector (Invitrogen) between Kpn I and EcoR I sites in which GFP is driven by a bidirectional cytomegalovirus promoter. Both constructs were verified by sequencing. The adenoviral GFP human KLF4 system was used as previously described by the Jain group (2, 24).

### Antibodies and flow cytometry

Prior to specific staining, cells (0.5 x 10^6^) were blocked with 5 µg/ml CD16/32 antibody for 5 min on ice. Following staining with specific antibody and respective controls, cells were analyzed on an LSR II flow cytometer (BD) as previously described (19, 21). For CD55, cells were incubated with 5 µg/ml of mouse anti-human CD55 mAb 2H6 followed by 10 µg/ml of Alexa 647 conjugated anti-mouse IgG.

### siRNA silencing of KLF4 and CD55

Flexitube siRNA Hs_KLF4_6 (Cat# SI03649191) and AllStars Neg. siRNA AF488 (Cat# 1027284) were purchased from QIAGEN Sciences, Inc. CD55 siRNA (Part# AM16708 ID145596) and silencer Cy 3-labeled negative control No.1 siRNA (Cat# AM4621) were purchased from ThermoFisher Scientific. Fifty to 100 nM of siRNA was delivered into HUVEC by Hiperfect transfect reagent (QIAGEN) following the manufacturer’s protocol. After 24 to 96 h of siRNA delivery, knockdown was confirmed by qPCR and FACS.

### Human CD55 promoter constructs

To append segments of the human CD55 promoter to a luciferase reporter, we amplified its proximal 120 bp (without KLF binding sites), the longer 190bp (containing the first pair of CACCC binding sites of KLF) and the full length 848 bp promoter sequence. All were amplified by PCR and each inserted into pGL3-Basic vector between Kpn I and Hind III sites. Each construct was verified by sequencing.

### Transfections and luciferase reporter assays

Luciferase pGL3- constructs were delivered into K562 cells using TransFast reagent (Promega) per the manufacturer’s instructions. After overnight incubation, transfected cells and a control were treated for 24 h with PMA and lysed using Reporter Lysis buffer (Promega). Luciferase activities were assayed using a FLUOstar OPTIMA (BMG Labtechnology) system.

### Chromatin IP

ChIP was performed using the human Exacta Chip kit (R&D Inc) according to the manufacturer’ manual. HUVEC or human aoric endothelial cells (HAEC) (Lonza) were cross-linked in log phase growth. KLF4 doublet sites in the CD55 promoter were identified using the NIH MatInspector of Genomatrix program and validated using the EPD bioinformatic promoter analysis tool. The primers used to amplify the KLF4 binding sequences in the CD55 promoter were: A) CD55CHIP1F 5’- CTGACCGCACCTCTGAC CACAAC -3’, CD55CHIP1R 5’- GCCGGAGCGAGTTGCAGTAAGCAG -3’, B) CD55CHIP2F 5’- GGCAGCAAGGCCTGCGATAC -3’, CD55CHIP2R 5’-GAGAGTGGGGAGGGGAAGGAG -3’, C) CD55CHIP3F 5’-CATACACACACGCACACTGGTG -3’, CD55CHIP3R 5’-CAGAGACCTGGG GAAACAGGTTG -3’. Negative control primers (located at CD55 intron 8) were F 5’-TGTTTGGCTAGCCTCCTGAT -3’; R 5’-AACGAAGTCAGGCATCTGCT -3’. Primers for eNOS: 5’-GGCTTGTTCCTGTCCATTGTGTA-3’, 5’-ATGTTACTGTGCGTCCACTCTGCT-3’. ChIP assays were conducted as previously described (2, 25, 26).

### Mice, murine aortic EC isolation, and CD4^+^ cell activation

All mice were maintained in the animal resource center of Case Western Reserve University using procedures conforming to the Institutional Animal Care and Use Committee (IACUC). *CD55^-/-^* mice (13) and both *KLF4^-/-^* and *KLF4-Tg* mice (2) were on the C57BL/6 background. Murine CD4^+^ T cells were purified from spleens and activated with anti-CD3/CD28 Dynabeads (Invitrogen, Cat# 114.52D) as previously described (14, 15). OT-II transgenic (Tg) CD4^+^ cells were incubated with 0.1 μg/ml ova_332-339_ peptide and WT, *KLF-4^–/–^*, or *KLF4-Tg* bone marrow (BM) derived macrophages isolated in each case by the CD11c purification kit (Miltenyi). Culture supernatants were assayed for IL-2 by ELISA (BD Biosciences). Murine peritoneal macrophages were prepared as previously described (14, 21). Primary murine ECs were isolated from aortas as previously described (19).

### Statistical analyses

Statistical significance for all experimental data was determined by Student’s T test (unpaired, two-tailed) performed in Microsoft Excel, SigmaPlot or GraphPad Prizm 6 with a significance threshold value of p<0.05. Except where indicated, all experiments were repeated 3 times in separate samples. Data are presented as mean values with SD.

## Results

### KLF4 augments EC CD55 gene transcription

**Figure 1:**
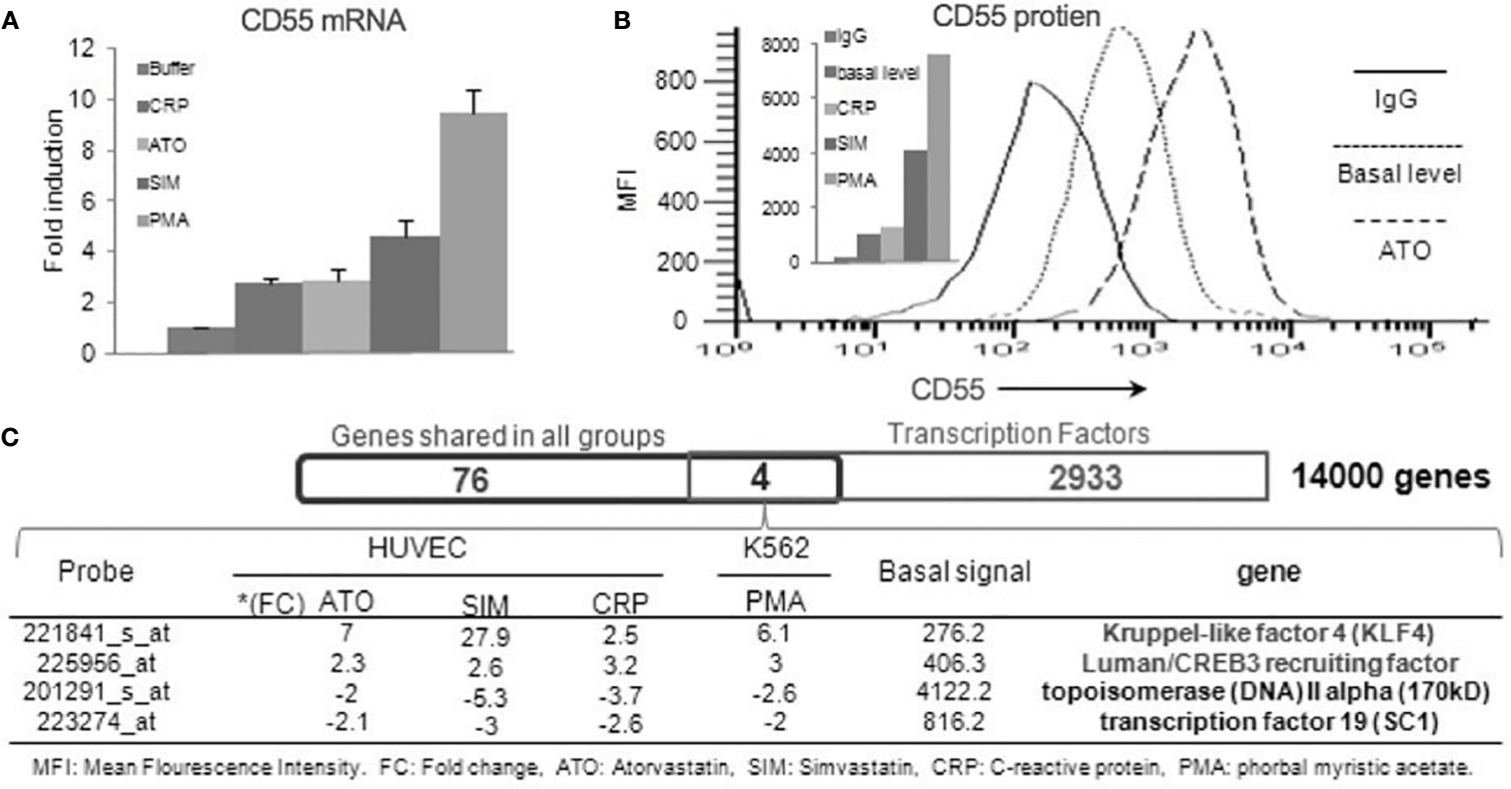
Human CD55 expression is upregulated by KLF4. **(A)** Confluent HUVEC were incubated with 20 nM of PMA, 8 μM of Simvastatin, 5 μM of Atorvastatin, 100 μg/ml CRP or media control and CD55 mRNA levels in the treated cells quantified by qPCR (n=3). **(B)** FACS analyses of the differentially treated HUVEC. A representative flow plot is shown for 48 h treatment with atorvastatin **(C)** RNA samples from the alternatively treated HUVEC in panel A were examined by Affymetrix gene array. 80 genes that changed by >2-fold up or down with all 4 treatments were overlapped with known transcription factors in the gene ontology database by Venn Diagram analysis. This identified (KLF4 and CREB3) that were upregulated and (Topoisomerase 2 and SC1) that were downregulated in response to all four treatments.

Prior studies of CD55 on ECs found that its expression is upregulated by phorbol myristate acetate (PMA) (27), C-reactive protein (CRP), and statins (28–31). Notably, the CD55 upregulation in response to statins was abolished by cycloheximide (CHX) or actinomycin-D, indicative of the requirement of the prior synthesis of another protein(s). To validate the extent to which each reported stimulus upregulates CD55, we incubated HUVEC with each for 24 h and measured CD55 mRNA and protein levels. All induced 2.5- to 10-fold increases in CD55 mRNA expression (**Figure 1A**) that translated into 2- to 7-fold increases in CD55 protein expression (**Figure 1B**). To determine whether the same or different mechanisms are involved in the upregulation by the different agents, we performed microarray assays of mRNAs induced by each agent.

Overlapping of the gene pools elicited by the different agents identified 80 genes with fold changes > 2 or <-2 that occurred in common to all. Interrogating the overlapped gene pool for known transcription factors identified four candidates (**Figure 1C**). Two (KLF4 and Luman/CREB2 recruiting factor) were associated with up-regulated CD55 expression and two (topoisomerase IV and SC1) with down-regulated CD55 expression. Of the two linked to up-regulated CD55 expression, KLF4 exhibited the most prominent augmentation. This finding that KLF4 controls CD55 expression in ECs would be in accordance with the finding (based on the presence of putative KLF binding sites) that it upregulates CD55 on IECs (22).

### Localization of the KLF4 responsive sites in the human CD55 promoter

**Figure 2:**
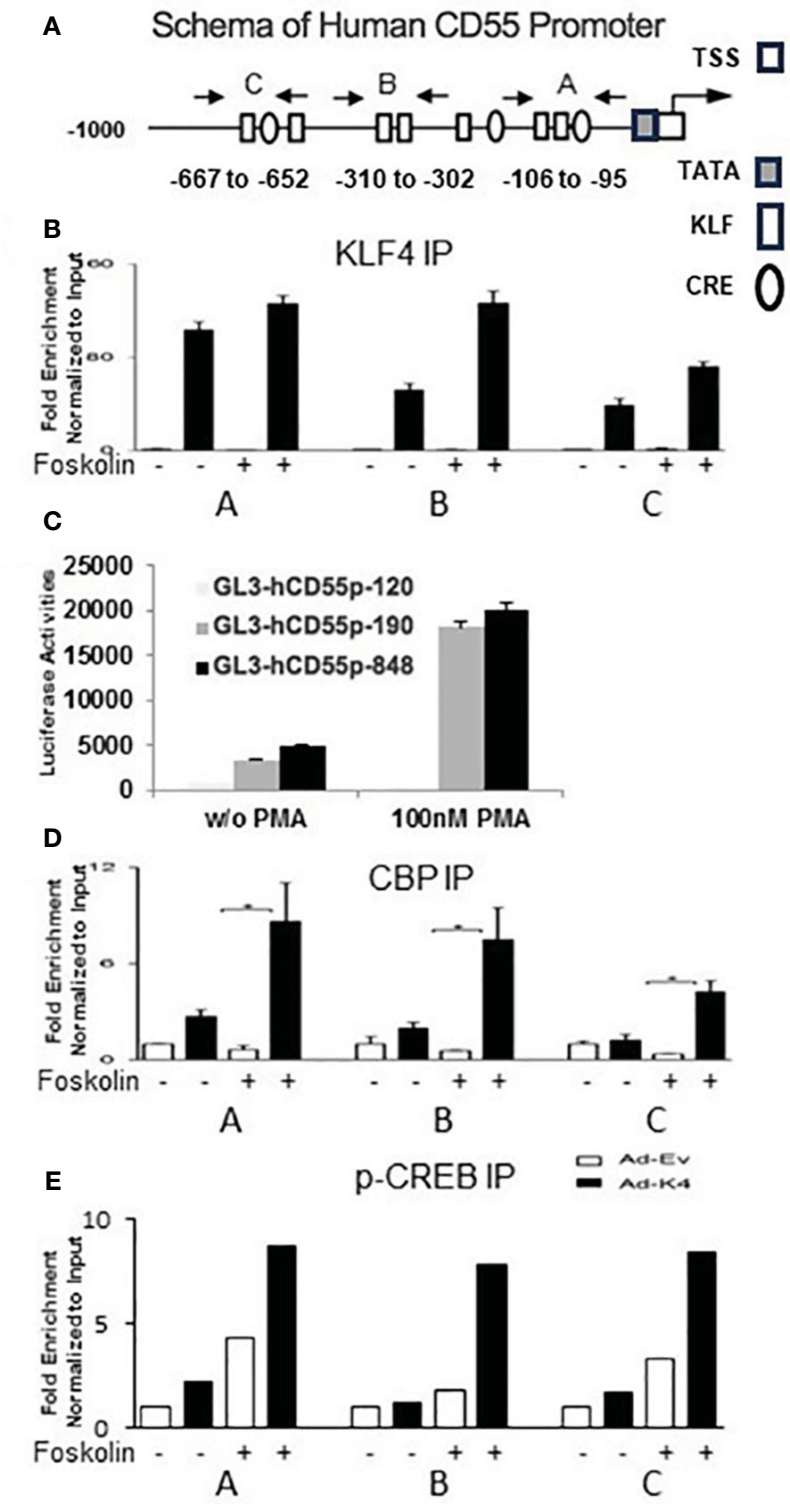
KLF4 regulates CD55 expression by binding to 3 paired CD55 promoter elements. **(A)** Schematic depiction of human CD55 promoter (1000 bp). KLF sites were identified by the MatInspector of Genomatrix program. They were confirmed by the EPD bioinformatic promoter analysis tool. The white rectangular boxes represent KLF binding sites (CACCC), the elliptic circles CRE sites, and white and dark square boxes the position of the TATA and TSS sequences. Three closely paired KLF binding sites; two adjacent to and one near canonical CRE consensus sequences are seen. The start site is located + 81 bp downstream of the TATA box (27). The arrows designate the primer pair positions for the paired KLF binding sites used in ChIP assays. **(B, D, E)** HUVEC were infected with adenovirus-KLF4 (Ad-K4) containing KLF4 or empty adenovirus-vector (Ad-Ev) control. Cells in log phase growth were treated for 30 min without or with 10 µM Forskolin, after which ChIP experiments were done with anti-human **(B)** KLF4, **(D)** anti-CBP, and **(E)** anti p-CREB antibodies. The white bars give the signal with Ad-EV and the black bars give the signal with Ad-KLF4 adenovirus. + or – designates the presence or absence of forskolin. **(C)** CD55 promoter luciferase constructs containing no KLF site (GL3-hCD55p-120), the first set of paired sites (GL3-hCD55p-190) and all three sets of paired KLF binding sites (GL3-hCD55p-848) were delivered into K562 cells. Transfection efficiency was ~ 15% After incubation for 24 h with 100 nM PMA, luciferase activities in cell lysates were measured (n=4). Data are given as the mean ± SEM.

The canonical cis sequence for the DNA of KLFs is CACCC (32). Usually, paired CACCC sites 2-20 bases apart are optimal for KLF binding. Analysis of the CD55 promoter revealed five potential paired KLF-binding sites at -106 to -95, -228 to -214, -310 to -302, -567 to -549 and -667 to -652 conforming to this motif with a confidence of p =.01. Three: -106 to -95, -310 to -302, and -667 to -652 were located nearby CRE sites, hereafter designated sites A-C (**Figure 2A**). To assess KLF4 occupancy at each site, we infected HUVEC with control (Ad-EV) or KLF4 (Ad-KLF4) adenovirus, stimulated the cells with vehicle or forskolin (known to upregulate KLF4), and performed ChIP at each site. All three sites showed KLF4 occupancy with marked enhancement by forskolin (**Figure 2B**). Site A played a predominant role in the KLF4 upregulation as a truncated 190 bp promoter construct containing only site A (GL3-hCD55p-190) showed nearly the same promoter activity (**Figure 2C**) as the extended 848 bp promoter construct possessing all three sites A-C (GL3-hCD55p-848). This predominance pertained both under basal conditions and following stimulation with PMA [which upregulates KLF4 as well as CD55 (**Figure 1A**)].

KLF4 transcriptional activity is dependent on recruitment of the coactivator CBP/p300 (2, 33), a process that requires the prior generation of p-CREB. In view of this, we correlated the positions of KLF4 sites with CRE sites in the CD55 promoter. All three KLF4 sites were adjacent to CRE sites (**Figure 2A**) prompting ChIP for p-CREB and CBP at each site. Marked p-CREB as well as CBP enrichment accompanied KLF4 enrichment at all three sites (**Figures 2D, E**). Less was observed in the absence of KLF4 but presence of forskolin (which upregulates adenylyl cyclase) and none in the absence of both. These studies pointed to KLF4/p-CREB/CBP occupancy of all three sites within the CD55 promoter participating in the CD55 upregulation. These data are consistent with findings in our earlier study that c-AMP analogs upregulate CD55 (27) and those in the previous study of KLF4 in IECs that prostaglandin-E2 (PG-E2) induction of protein kinase A (PKA) upregulates CD55 (22). The ChIP assays herein highlight the close spatial juxtaposition of p-CREB and CBP next to KLF4 in the CD55 promoter.

### KLF4 upregulates CD55 in multiple cell lineages, its upregulation precedes that of CD55, and CD55 and KLF4 function are correlated with each other

**Figure 3:**
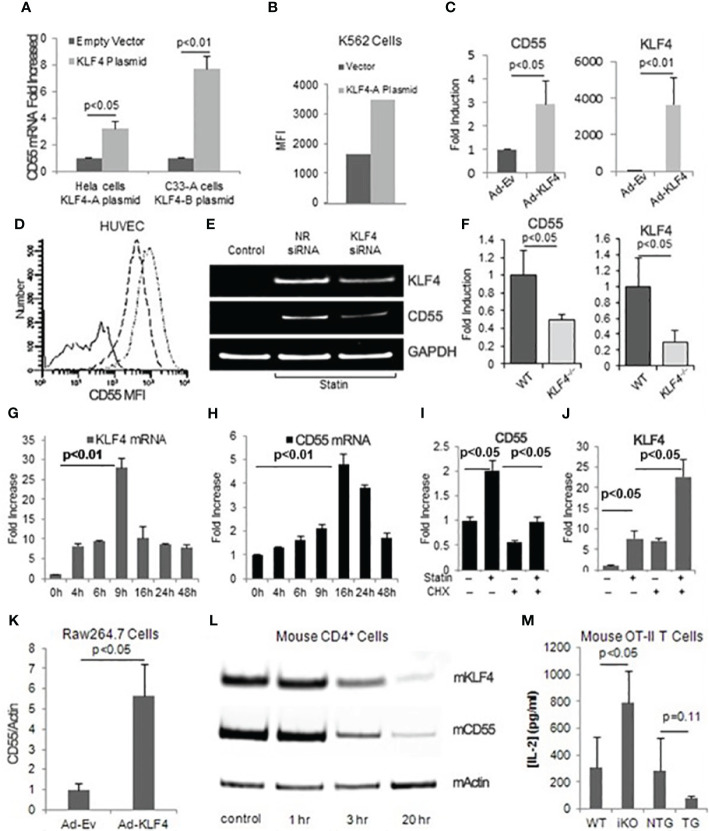
KLF4 regulates CD55 expression in multiple cell types and its upregulation precedes that of CD55. **(A)** HeLa cells transfected with pTracer EF/V5 His C-KLF4 (KLF4-A) and C33-A cells transfected with pEGFP-N1-KLF4 (KLF4-B) were incubated for 48 h, after which RNA prepared from GFP positive cells was assayed for CD55 transcripts (n=3). **(B)** K562 cells were transiently transfected with KLF4-A for 68 h, after which surface CD55 levels (Mean channel fluorescence intensity MFI) of GFP positive cells were measured. **(C)** HUVEC were infected with KLF4 adenovirus or empty adenovirus after which CD55 and KLF4 mRNA levels were quantified by qPCR (n=3). The data were normalized to GAPDH levels. **(D)** HUVEC infected with KLF4 or control adenovirus were incubated for 48 h, after which CD55 expression levels were quantitated by FACS. The solid line represents control IgG; the dash line control virus infected cells stained for CD55; and the dash-dot line KLF4 adenovirus infected cells stained for CD55. **(E)** HUVEC pretreated with 5 μM Atorvastatin were transfected with KLF4 siRNA. 48 h later, KLF4 and CD55 mRNA levels were quantitated. >95% of the siRNA treated cells retained viability and remained green at each time point. The band of KLF4 in untreated cells is difficult to see as it is up to 30-fold lower as shown panel 3E. **(F)** CD55 and KLF4 mRNA levels were quantitated in primary aortic ECs from wild type and mice conditionally deficient in EC KLF4. Fold changes were normalized to β-Actin levels (n=3). **(G, H)** Simvastatin (8 μM) was added to cultures of confluent HUVEC after which KLF4 **(G)** and CD55 **(H)** mRNA expression levels were quantified at increasing times (n=3). **(I, J)** HUVEC were pre-incubated for 1 h without or with cycloheximide (100 μM), after which the alternatively treated cells were incubated for 24 h without or with atorvastatin (5 μM) and CD55 **(I)** and KLF4 **(J)** mRNA levels were assayed. Data were normalized to GAPDH (n=3). **(K)** Murine RAW264.7 macrophages were infected for 24 h with KLF4 or control adenovirus, after which KLF4 and CD55 mRNA expression were quantified by qPCR. Data normalized to β-Actin (n=4). **(L)** Splenic CD4^+^ cells were incubated for 72 h with anti-CD3/CD28 Dynabeads, after which the cells were assayed for *KLF4* and *CD55* mRNA levels at the indicated time points. β-Actin loading controls are shown **(M)** WT, *KFL4^–/–^* (iKO - inducible knockout), KLF4-Tg (TG - KLF4 transgene) or NTG (KLF4 transgenic related background control mouse) macrophages (each 1x10^5^) were incubated with 0.1 μg/ml ova_332-339_ ova specific OT-II transgenic CD4^+^ cells (1x10^6^) for 72 h after which culture supernatants were assayed for IL-2 by ELISA (n=3).

To assess the effects of KLF4 expression on CD55 upregulation in different cell types, we delivered KLF4 to HeLa cells, K562 cells, and HUVEC using KLF4 cDNA linked to GFP. qPCR of sorted GFP^+^ cells showed that KLF4 up-regulated CD55 mRNA levels 3.2-fold in Hela cells (**Figure 3A** left side), 7.7 -fold in the human cervical cancer cell line (C33-A) (**Figure 3A** right side) and 3-fold in HUVEC (**Figure 3C**). Silencing of KLF4 in statin treated HUVEC attenuated the statin up-regulation of CD55 consistent with KLF4 being the cycloheximide sensitive precursor protein needed for the statin up-regulation of CD55 (**Figure 3E**). In ECs of mice selectively deficient in *KLF4*, the CD55 mRNA expression level was 50% lower than in ECs of WT mice (**Figure 3F**), validating the dependence of CD55 expression on KLF4 *in vivo*. Taken together, the results indicated that KLF4 regulates CD55 gene transcription in epithelial, myeloid, and ECs, and that the up-regulation of CD55 that occurs with statins is mediated by KLF4.

We next examined how statin induced up-regulation of KLF4 and that of CD55 are temporally related. Following simvastatin addition to confluent HUVEC, KLF4 mRNA up-regulation (**Figure 3G**) occurred 6-8 h earlier than CD55 mRNA up-regulation (**Figure 3H**). Comparable results were obtained with atorvastatin (not shown). Cycloheximide abolished the CD55 up-regulation consistent with the CD55 upregulation occurring as a result of prior upregulation of KLF4 protein (**Figure 3I**). Unexpectedly, the cycloheximide pretreatment upregulated KLF4 mRNA expression 4-5-fold over statin alone indicating that KLF4 expression itself is regulated by the prior synthesis of one or more other factors (repressors) (**Figure 3J**), an issue that will be addressed below. KLF4 has been linked with inhibition of cell growth. In support of upregulated CD55 potentially being involved in the KLF4 growth inhibitory effect, the microarray assays of CD55 upregulated cells (**Figures 1A, B**) showed down-regulation of multiple genes associated with cell cycle progression (**Supplementary Table I**).

To determine whether KLF4 participates in modulating adaptive T cell responses, we infected the Raw246.7 murine macrophage line with Ad-(murine) KLF4-GFP and assayed CD55 mRNA levels. As observed with the various human cell types above, the Raw246.7 cells that received Ad-KLF4 (but not Ad-EV) up-regulated CD55 mRNA expression levels 7-fold (**Figure 3K**). CD4^+^ T cell IL-2, IFN-γ, and TNF-α contribute to atherothrombosis as well as other inflammatory conditions and our previous studies (14) showed that lifted CD55 restraint on autocrine C3ar1/C5ar1 signaling by DC-CD4^+^ cell partners drives Teff responses. Because of this, we next investigated whether changes in KLF4 levels occur in immune cells during activation and whether the changes are correlated with those of CD55. KLF4 down-regulation in activated CD4^+^ splenocytes paralleled down-regulation of CD55 mRNA expression (**Figure 3L**). In another experiment, we incubated murine *KLF4^-/-^* or *KLF4* transgenic (overexpressing) macrophages or WT controls with ova_323-339_ and (ova specific) OT-II transgenic CD4^+^ cells, after which we quantified IL-2 production. As we previously reported for reduced CD55 expression in antigen presenting macrophages (14, 21), reduced KLF4 expression accompanied augmented OT-II cell IL-2 production, whereas KLF4 overexpression accompanied decreases in IL-2 (**Figure 3M**).

### Anti-inflammatory and anti-thrombotic effects of KLF4 are dependent on upregulated CD55

**Figure 4:**
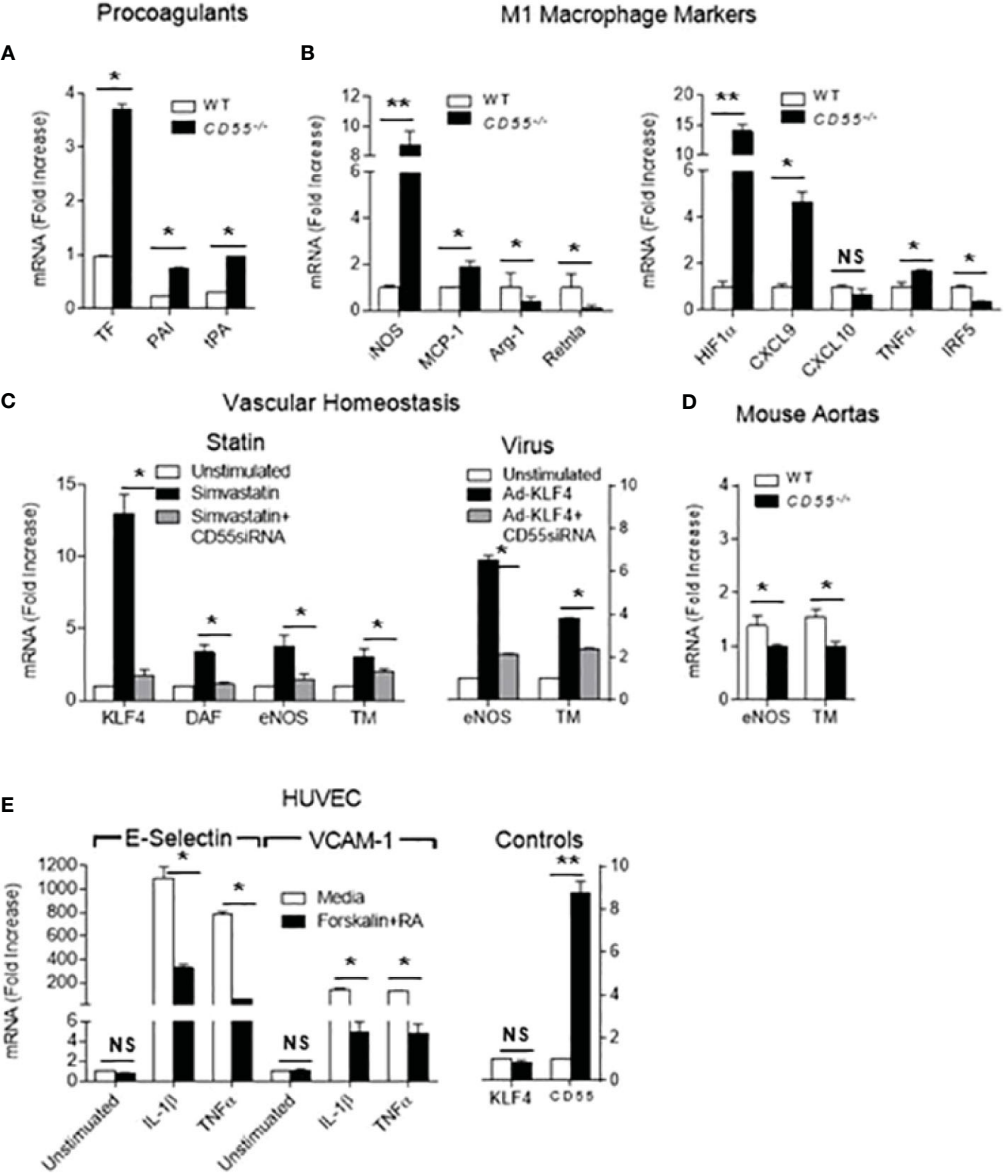
Many cardioprotective effects of KLF4 depend on upregulation of CD55. **(A)** Primary cultured aortic ECs from WT and *CD55^-/-^* mice were extracted with Trizol and levels of tissue factor (TF), plasminogen activator inhibitor (PAI) and tissue plasminogen activator (tPA) mRNA transcripts in total RNA were quantified. **(B)** Peritoneal macrophages were obtained from WT and *CD55^-/-^* mice pretreated i.p. for 48 h with thioglycollate. Trizol extracts of the WT and *CD55^-/-^* macrophages were assayed for MCP-1, iNOS, Arg-1, and Retnla (left panel) as well as HIF1α, CXCL9, CXCL10, TNFα, and IRF5 (right panel) by qPCR. **(C)** Untreated HUVEC or HUVEC pretreated with *CD55^-/-^* siRNA were incubated for 24 h with 8 uM simvastatin (left panel) and CD55, KLF4, eNOS, and TM mRNA levels quantitated by qPCR. qPCR and FACS documented that CD55 was silenced and absent. In a parallel experiment (right panel), untreated HUVEC and *CD55^-/-^* sRNA treated HUVEC were infected with KLF4-Adenovirus after which the cells were incubated for 24 h with simvastatin and eNOS and TM mRNA levels quantified. The assays in cells that did not receive KLF4 virus documented that KLF4 exerted its characteristic functions. **(D)** Perfused aortas from WT and *CD55^-/-^* mice were extracted with Trizol and mRNA levels of eNOS and TM were assayed by qPCR. **(E)** HUVEC were incubated for 24 h with IL-1-β (1 ng/ml) or TNF-α (1 ng/ml) in the absence of presence of forskolin (10 ng/ml) and C3ar1-A/C5ar1-A (10 ng/ml each). Trizol extracts of the cells were then assayed for E-Selectin mRNA levels by qPCR. qPCR for KLF4 and CD55 mRNA levels showed no change in KLF4 expression levels but upregulation of CD55 mRNA levels. * = < .05, ** = < .005. NS, Not Significant.

We next examined whether the vasculo-protective effects of KLF4 upregulation are tied to CD55 upregulation. We first compared the expression of KLF4 dependent genes in primary aortic ECs derived from WT *vs CD55^-/-^* mice. CD55 deficiency not only negated KLF4 downregulation of tissue factor, PAI and tPA, but conversely upregulated each pro-thrombotic protein (**Figure 4A**). We then assessed KLF4 dependent genes in *CD55^-/-^* macrophages. As found for EC pro-coagulant proteins, macrophage CD55 deficiency not only prevented KLF4 downregulation of proinflammatory MCP-1 and iNOS, but conversely upregulated both proteins. It similarly reversed KLF4 up-regulation of anti-inflammatory Arg-1 and Retnla (**Figure 4B** left). A comparable pattern was observed for macrophage HIF-1α, CXCL9, CXCL10, TNF-α, and IRF5 (**Figure 4B** right).

We next examined the effect in human ECs, i.e., HUVEC, of CD55 knockdown on anti-thrombotic genes upregulated by KLF4. Silencing CD55 markely reduced KLF4 dependent expression of homeostatic iNOS and TM when KLF4 was physiologically upregulated by statin addition. It significantly reduced their expression when KLF4 was overexpressed by KLF4-Ad (**Figure 4C**). Notably, under conditions of statin upregulation of KLF4, silencing of CD55 markedly reduced KLF4 mRNA expression, an issue that will be further addressed below. Analyses of freshly isolated aortas showed that TM and eNOS mRNA levels were similarly repressed in *CD55^-/-^* mice compared to WTs (**Figure 4D**) arguing that this dependence applies *in vivo*. We next tested whether KLF4 repression of VCAM-1 and E-Selectin expression on ECs in response to TNF-α and IL-1β is likewise dependent on CD55. Forskolin plus C3ar1/C5ar1 antagonism, both of which upregulate CD55 (15, 27) suppressed TNF-α or IL-1β induction of VCAM-1 and E-Selectin in ECs (**Figure 4E**) in a fashion similar to KLF4. These data argued that many antithrombotic and immunosuppressive effects of KLF4 expression may be mechanistically interconnected with upregulation of CD55.

### The dependence of KLF4 cardioprotective effects on CD55 is due to CD55’s involvement in recruiting CBP

Several past studies have linked KLF4’s immunosuppressive function to its out-competition with NF-kB for CBP, but the mechanism of this process has remained obscure (2, 34–36). **Figure 2** showed that both CREB and CBP were present by ChIP at all three KLF4 sites in the CD55 promoter. **Figure 3** showed that the effects of CD55 and KLF4 up and down-regulation paralleled each other, and **Figure 4** showed that KLF4 function is dependent on CD55.

**Figure 5:**
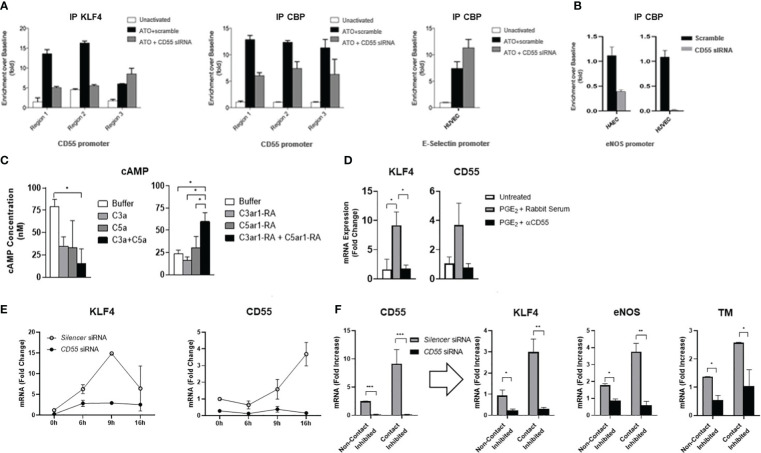
CD55 is essential for KLF4 recruitment of CBP and KLF4 expression. **(A)** HUVEC were transfected with siRNA targeting CD55 or scramble control for 6 days. After confirming CD55 expression was knocked down (not shown), HUVEC were stimulated with atorvastatin (ATO) for 24 hr after which they were cross linked and harvested for chromatin. Chromatin IP experiments were done using the anti-human KLF4 and anti-CBP Abs as in **Figure 2B**. After ChIP, samples were assayed for KLF4 and CBP enrichment at the 3 regions of the CD55 promoter while additionally assaying the promoter of E-selectin for CBP enrichment by qPCR. **(B)** The experiment in Figure **(A)** was repeated this time using both HAEC and HUVEC and assaying for CBP association with the eNOS promoter. **(C)** Activity of cAMP in HUVEC for 10 min with forskolin (30 μM) alone (Buffer) or, left, with C3a (100 ng/ml) or C5a (100 ng/ml) or both or, right, with C3ar1 antagonist (30 nM) or C5ar1 antagonist (30 nM) or both. **(D)** HUVEC were stimulated with PGE_2_ (100 µM) for 4 h in the absence or presence of anti-CD55 blocking Ab and KLF4 and CD55 mRNA levels quantified by qPCR. **(E)** HUVEC were pre-transfected with siRNA targeting CD55 or with scrambled control for 8 days. Atorvastatin (5 μM) was added to cultures after which KLF4 and CD55 mRNA levels were quantified at increasing times. **(F)** HUVEC were pre-transfected with siRNA targeting CD55 or with scrambled control for 6 days. HUVEC were then maintained under either confluent or sub-confluent conditions, and CD55, KLF4, eNOS, and TM mRNA levels were quantitated by qPCR.

To gain mechanistic insight, we first tested whether CD55 is needed for KLF4 recruitment of CBP. We treated HUVEC with CD55 or scrambled siRNAs. After confirming CD55 protein knockdown in the CD55 siRNA but not scrambled siRNA treated cells, we incubated the alternatively treated HUVEC with atorvastatin for 24 h. We then performed ChIP with anti-KLF4 and anti-CBP and compared the binding of CBP to the CD55 and E-selectin promoters. In the scrambled siRNA treated HUVEC, atorvastatin treatment caused increases of KLF4 and of CBP at all three KLF4 sites in the CD55 promoter, albeit less to site C (**Figure 5A**). In HUVEC silenced in CD55, the atorvastatin treatment led to less CBP at all three sites as well as less recruitment of KLF4 at sites A and B (**Figure 5A** left). Conversely, an increase in CBP was observable in the E-selectin promoter (**Figure 5A** right). This result prompted studies of the effect of CD55 knockdown on CBP recruitment to another extragenic promoter, i.e. the eNOS promoter. Both HAEC and HUVEC treated with scrambled siRNA showed strong CBP association with the eNOS promoter. In contrast, both cell types silenced in CD55 showed markedly reduced or no CBP association with eNOS (**Figure 5B**).

The recruitment of CBP requires the prior presence at CRE sites of p-CREB nearby promoter bound KLF4. Since C3ar1 and C5ar1 are Gi coupled GPCRs that repress adenylyl cyclase activation (needed for p-CREB generation), and upregulated CD55 de-represses this Gi activation, we next investigated the effects in HUVEC of C3ar1 and C5ar1 signaling *vs* the blockade of each on activation of the adenylyl cyclase product, cyclic AMP (c-AMP). Signaling through each GPCR inhibited c-AMP activation, while signaling through both additively inhibited the c-AMP activation (**Figure 5C** left). Conversely, pharmaceutical blockade of each GPCR had the opposite effects (**Figure 5C** right). CD55 blockade abolished augmentation of KLF4’s upregulatory effect on CD55 by PGE2, the process previously ascribed to p-CREB augmentation of KLF4 function in IECs (**Figure 5D**). The findings that repression of autocrine C3ar1/C5ar1 signaling is needed for c-AMP activation, a function of upregulated CD55, thus explains the dependence of p-CREB generation and recruitment of CBP on upregulation of CD55 expression. Since this process operates commonly in different cell types, the findings provide a mechanism that, in principle, would not on apply to multiple KLF4 regulated genes in the same cell type but also to cells of different lineages.

### KLF4 expression depends on CD55 expression

**Figure 6:**
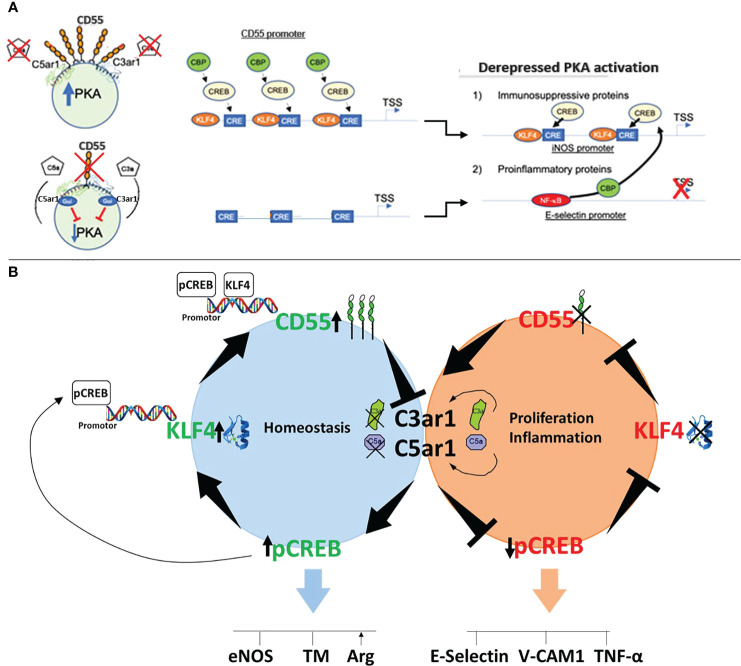
Mechanism of CD55 control of KLF4 expression and function. **(A)** Schematic of the mechanism by which KLF4 upregulation of CD55 enables KLF4 recruitment of CBP. The sequential panels show that upregulation of KLF4 by a KLF4 stimulus, e.g. statin, upregulates CD55. The upregulated CD55 suppresses autocrine C3ar1/C5ar1 signaling that enables PKA generation of p-CREB. The p-CREB recruits CBP and in combination with KLF4 enables p-CREB and CBP to the promoter of eNOS and other KLF4 target genes while reciprocally depriving the promoters of E-Selectin and other proinflammatory genes of the CBP coactivator. **(B)** Cartoon of how CD55 expression regulates KLF4 expression. Left: When CD55 is present C3ar1/C5ar1 signaling is suppressed. As a result, PKA generation of p-CREB occurs. The obligate participation of p-CREB for KLF4 transcription upregulates KLF4 expression. Upregulated KLF4 expression upregulates CD55 expression. Upregulated CD55 expression sustains repression of C3ar1/C5ar1 signaling. Right, when CD55 is absent the opposite sequence of events occurs.

The findings in HUVEC silenced in CD55 that KLF4 expression was reduced (**Figure 4C**) raised the possibility that KLF4 expression itself may depend on CD55. To formally test this possibility, we performed two complementary experiments. 1) We first re-examined the kinetics of KLF4 and CD55 expression in statin treated HUVEC pre-silenced in CD55. Statin addition to untreated HUVEC (**Figures 3G, H**) had shown that upregulation of KLF4 precedes upregulation of CD55 by 7 hr. Kinetic studies in HUVEC silenced in CD55 showed that in contrast to scrambled siRNA treated HUVEC in which the sequential upregulations paralleled those in untreated HUVEC (**Figures 3G, H**), no upregulation of KLF4 occurred in the HUVEC pre-silenced in CD55 (**Figure 5E**). 2) We next assayed HUVEC silenced in CD55 for the upregulation of KLF4 and attendant homeostatic proteins that accompanies the transition of ECs from conditions of growth to conditions of confluence. Other studies in our group (37) showed that CD55 expression upregulates in ECs upon transition from sub-confluence to confluence. While HUVEC transfected with scrambled siRNA showed marked upregulation of KLF4, eNOS and TM with contact inhibition, HUVEC silenced in CD55 showed essentially no upregulation KLF4 or of either of the homeostatic proteins (**Figure 5F**). The changes observed in this experiment were independent of a drug (e.g. statins) or other exogenous intervention. As found in **Figure 3** that KLF4 knockdown abolished the expression of CD55, these studies in two independent settings (**Figures 5E, F**) showed that CD55 knockdown abolished the expression of KLF4. Despite viral expression of KLF4 in ECs with Ad-KLF4 (**Figure 3A**), CD55 siRNA knockdown abolished KLF4’s capacity to transactivate homeostatic TM and eNOS (**Figure 4C**). In statin treated ECs CD55 deficiency disabled KLF4’s transfer of CBP from proinflammatory E-selectin to homeostatic eNOS (**Figures 5A, B**). These findings taken together with the other studies in this manuscript thus indicate that KLF4’s and CD55’s functions are interconnected. Studies *in vivo* in murine models and human samples that will be presented in the Discussion are in line with this proposition. A diagram summarizing the interrelationship found in this study between KLF4 and CD55 functions in ECs and macrophages is given in **Figure 6A**.

The mechanism underlying the dependence of KLF4 function on CD55 expression is that KLF4 function depends on the transcription factors p-CREB and CBP (33, 38, 39), both of which are dependent on CD55 inhibition of autocrine C3ar1/C5ar1 signaling (Diagrammed in **Figure 6B**). The global heightening of p-CREB enabled by upregulated CD55, in principle, allows multiple, if not many, KLF4 regulated genes to recruit the essential coactivator CBP/p300 thereby conferring KLF4 transactivation. Evidence in the literature indicates that p-CREB and CBP additionally promote KLF4 expression via binding to the KLF4 promoter (39), the KLF4 enhancer (40) or both. The data herein taken together with that finding thus argue that KLF4 and CD55 are intimately interconnected in that the expression and function of each depend on the other.

## Discussion

Much data (2, 25, 26, 41) have shown that KLF4 evokes an immunosuppressive, anti-inflammatory, and antithrombotic phenotype in both ECs and macrophages. Consequently, KLF4 confers cardioprotective effects that attenuate atherosclerosis/atherothrombosis and raise the threshold for autoimmune and other inflammatory diseases. While KLF4 induces the transcription of many anti-inflammatory and anti-thrombotic proteins in both ECs and macrophages, as well as other immune cell types via a CBP dependent process, the cellular mechanism that enables this process has remained unclarified. This study shows that upregulation of CD55 protein and its restraint on autocrine C3ar1/C5ar1 signaling implements these KLF4 processes in each cell type. Remarkably, the data herein additionally show that the expression of KLF4 itself is dependent on CD55 expression.

The results of the studies herein explain several past *in vivo* observations. An analysis of glomerular mRNA from renal transplant patients that developed thrombotic microangiopathy (TMA) found complement activation and increased PAI-1 expression that occurred in association with reduced KLF4 and reduced CD55 (42). Notably, the sister human membrane associated complement regulators CD46 (aka membrane cofactor protein) and CD59 (membrane inhibitor of reactive lysis) were increased. Another study reported that expression of TF and Von Willibrand factor (VWF) by ECs, two prothrombotic proteins repressed by KLF4, was increased in the absence of CD55 (43, 44). In line with these observations in human tissues, studies of glomerular ECs in murine models of TMA found that KLF4 and CD55 were concurrently reduced in conjunction with increased deposition of C3 (45). Again, expression of the corresponding murine cellular regulators, CR1 related protein Y (Crry) and CD59 were increased. In-line with these studies, studies of murine atherosclerosis by ourselves (*Medof ME et al. unpublished*) have found that *CD55^-/-^* mice develop heightened atherosclerotic and thrombotic changes closely corresponding to those of *KLF4^-/-^* mice.

Suppression of autocrine C3ar1/C5ar1 signaling by CD55 enables upregulation of adenylyl-cyclase generation (15), c-AMP production, and consequent PKA phosphorylation of CREB. The p-CREB binds to CRE sites in cis sequences nearby or remote from KLF4 sites and allows recruitment of CBP (or p300). In CD55’s absence, C3ar1/C5ar1 signaling is potentiated, adenylyl cyclase is downregulated, and p-CREB generation is repressed. Recruitment of CBP to p-CREB connected with promoter bound KLF4 does not occur and KLF4 immunosuppression is disabled. Consistent with the findings herein regarding KLF4 regulation of immunosuppressive and antithrombotic genes are findings that expression of anti-atherogenic apolipoprotein A in macrophages requires joint binding of KLF4 and p-CREB to the apolipoprotein A promoter (34).

According to available data, cell endogenous complement production (46) and autocrine C3ar1/C5ar1 signaling operate in most if not all cell types (14, 15, 19, 20). C3ar1/C5ar1 transduction provides viability signals to immune cells, ECs, and other cardiovascular relevant cell types under homeostatic conditions (14, 19, 20). The tonic production of C3a/C5a and its consequent C3ar1/C5ar1 transduction and coordinate IL-6R-STAT3 signaling involved in viability signaling (19, 20) may be one source of the cycloheximide sensitive repression of KLF4 expression that we observed in our studies of HUVEC treated with this protein synthesis inhibitor.

Amplification of autocrine C3ar1/C5ar1 signaling in immune cells and ECs evoked by CD55 downregulation induces proinflammatory cytokine production and promotes mitotic function. In contrast, repression of this GPCR signaling evoked by CD55 upregulation induces autocrine TGF-β production, Treg induction (15) and monocyte and EC growth restraint (19). The significant finding in this study that repression of C3ar1/C5ar1 signaling by upregulated CD55 is required for expression of KLF4 together with the finding that KLF4 is required for expression of CD55 indicate that KLF4 and CD55 expression and function are intimately tied to each other. It could be argued that the function of KLF4 is to upregulate CD55 that represses autocrine C3ar1/C5ar1 signaling. It alternatively could be argued that the function of CD55 is to both induce KLF4 expression and carry out the processes connected with it. The data herein show that both proteins functioning coordinately are needed to enable KLF4 transactivation mediated elaboration of its immunosuppressive and anti-thrombotic activities.

A prior study (33) provided evidence that a critical function of CBP association with KLF4 is that its binding induces KLF4 and H4 histone acetylation needed to enable KLF4 transcription as well as enable KLF4’s transactivation of KLF4 target genes. That study further showed that under pro-inflammatory/growth conditions as opposed to immunosuppressive conditions, the histone deacetylase H3 (HDAC3) rather than CBP/p300 associates with KLF4. These effects of CBP association with KLF4 may be the main reason for the dependence of KLF4 transcription on CBP recruitment. While our findings regarding the requirement of CD55 for p-CREB and CBP recruitment to KLF4 raise the possibility that KLF4 and histone deacetylation also are dependent on CD55, further studies will be needed to directly test this. Further studies likewise will be needed to test if HDAC3 epigenetic regulation which opposes KLF4 transcription is interconnected with lifted CD55 restraint of C3ar1/C5ar1 signaling.

The finding that p-CREB recruitment of CBP is dependent on CD55 also raises the question of whether the binding of other coactivators to KLFs, e.g. CRTC2/3 (39) or other KLFs similarly are regulated by CD55. In addition, our observation that the binding of KLF4 to the eNOS promoter is reduced by silencing CD55 expression also raises the question of whether p-CREB and/or CBP not only augment transcription of KLF4 target genes, but also function to enhance KLF4 binding to KLF4 promoter sequences. In support of the latter possibility, structural studies of erythroid Kruppel like factor (EKLF) have shown that its minimal transactivation domain (TAD) that corresponds to that in KLF2, KLF4, KLF5, and KLF15 binds to four domains in CBP/p300 (47).

While CD55 lifts dominant C3ar1/C5ar1 Gαi restraint on adenylyl cyclase activation, a concurrent Gαs signal is needed to induce adenylyl cyclase activation. Studies by others (48) of Treg induction and ourselves (15) of accompanying CD55 upregulation needed for p-CREB binding to the Foxp3 promoter have found that the Gαs signal is provided by A2a receptor (A2aR) signaling induced by adenosine produced by CD4^+^ cell associated CD39 and CD73 conversion of c-AMP. Studies by others (22) have shown that the Gαs signal can be provided by PGE2 transduction through Gαs coupled EP4 receptors. Our data showed that PGE2 induced upregulation of KLF4 expression requires suppression of autocrine C3ar1/C5ar1 signaling by CD55. Upregulated generation of p-CREB by PGE2 was found to be capable of promoting upregulation CD55 in the earlier study of KLF4, but the effects of the CD55 upregulation were attributed to protection of mucosal cells from systemic complement attack, the function originally associated with CD55 (11). An endogenous cellular role of CD55 in regulating KLF4 expression or in conferring KLF4’s functions was not envisioned.

The findings herein that KLF4 expression is regulated by CD55 and that CD55 and KLF4 expression are coordinated are relevant to the biology of nitric oxide. They show that the absence of CD55 in macrophages abolishes KLF4’s down-regulatory effects on iNOS and up-regulatory effects on Arg-1. They likewise show that the absence of CD55 in ECs reverses KLF4’s up regulatory effects on eNOS. These processes uniformly have been attributed to KLF4 “regulation” without mechanistic underpinning. In support of the findings herein constituting that mechanism, previous studies (49, 50) have shown CD55 deficiency in paroxysmal nocturnal hemoglobinuria (PNH) is associated with free nitric oxide and that expression of CD55 is decreased, like that of KLF4, on iNOS^+^ peripheral blood leukocytes in patients with type II diabetes.

In view of the central involvement of ECs and immune cells in a broad spectrum of disease states, the KLF4-CD55 connection likely has broad implications for acute and chronic inflammatory states. Reports over many years have pointed to the involvement of complement in cytokine production (51), adaptive T cell responses (52), cell viability/growth, and tissue repair (53), as well as stem cell recruitment and differentiation (52, 54). The effects in all cases were attributed to “crosstalk” between plasma derived complement activation fragments and cells. The new insights on the critical role that CD55 regulation of autocrine C3ar1/C5ar1 signaling plays in cell biological processes, now applicable to ECs, SMCs, myeloid cells, and fibroblasts (19, 20) may account for many of these observations. Given that KLF4 expression has been shown to modulate cell growth/tumorigenesis, viability, tissue repair, as well as stem cell recruitment and differentiation, more studies will be needed to determine if CD55’s suppression of C3ar1/C5ar1 signaling is interconnected with some or many of these effects. The data herein provide a framework for systematic testing of these KLF4 associations.

## Data availability statement

The data presented in the study are deposited in the Gene Expression Omnibus repository, accession number GSE255080.

## Ethics statement

The animal study was approved by Case Western Reserve University IACUC. The study was conducted in accordance with the local legislation and institutional requirements.

## Author contributions

FA: Formal Analysis, Investigation, Methodology, Project administration, Validation, Writing – review & editing. GZ: Investigation, Methodology, Software, Writing – review & editing. MH: Investigation, Methodology, Writing – review & editing. WH: Investigation, Methodology, Software, Writing – review & editing. MS: Investigation, Methodology, Supervision, Writing – review & editing. MJ: Formal Analysis, Resources, Supervision, Validation, Writing – review & editing. MM: Conceptualization, Funding acquisition, Project administration, Resources, Supervision, Writing – original draft, Writing – review & editing.
