# Supplementary material for: KLF4 and CD55 expression and function depend on each other
Source: Front Immunol. 2024 Feb 9;14:1290684. doi: 10.3389/fimmu.2023.1290684 (PMC10884306; doi:10.3389/fimmu.2023.1290684)
Supplement: Supplementary file 1 [file Table_1.docx]

**Supplementary Table 1**

| Probe | K562-FC | PMA-FC | CRP-FC | ATO-FC | SIM-FC | gene name |
| --- | --- | --- | --- | --- | --- | --- |
| 209891_at | -3 | -4.3 | -5.7 | -2.8 | -8 | AD024 |
| 228323_at | -2 | -2.6 | -6.1 | -2.3 | -2.6 | AF15Q14 |
| 209464_at | -3.7 | -3.5 | -2.5 | -2.3 | -4.9 | Aik2 |
| 203755_at | -3 | -2.8 | -3.5 | -2.5 | -4.6 | BUB1B |
| 210052_s_at | -2.3 | -2.8 | -3.2 | -2.1 | -2.5 | C20ORF1 |
| 203418_at | -4.3 | -3 | -2.5 | -2 | -3.7 | CCNA2 |
| 214710_s_at | -3 | -2.1 | -3.2 | -2 | -2.6 | CCNB1 |
| 210559_s_at | -2.1 | -4.9 | -2.1 | -2.6 | -4 | CDC2 |
| 202870_s_at | -2.5 | -2.8 | -3.5 | -2.1 | -2.8 | CDC20 |
| 204962_s_at | -4.6 | -4 | -4.9 | -2 | -6.5 | CENPA |
| 205046_at | -2.3 | -3.7 | -3.7 | -2.3 | -2.1 | CENPE |
| 203145_at | -2.3 | -2 | -3 | -2 | -2.8 | DEEPEST |
| 202532_s_at | -3.5 | -2.5 | -2.6 | -2 | -2.3 | DHFR |
| 234863_x_at | -2.6 | -2.8 | -2.3 | -2.5 | -9.8 | FBXO5 |
| 204318_s_at | -2.5 | -3.5 | -5.7 | -2.3 | -4.9 | GTSE1 |
| 218663_at | -2.8 | -2.8 | -6.1 | -2.1 | -3 | HCAPG |
| 212949_at | -5.3 | -4.3 | -3.5 | -3.5 | -2.5 | HCAPH |
| 219306_at | -3.7 | -3 | -4.6 | -2.1 | -3.2 | hklp2 |
| 204444_at | -3.5 | -3.5 | -5.3 | -2 | -3.5 | KNSL1 |
| 204709_s_at | -3.2 | -4.3 | -4.9 | -2.3 | -3.2 | KNSL5 |
| 218585_s_at | -3 | -2 | -2.3 | -4 | -4.3 | L2DTL |
| 220651_s_at | -3.2 | -2.3 | -2.5 | -5.7 | -19.7 | MCM10 |
| 222037_at | -3.7 | -2.5 | -2 | -5.7 | -5.3 | MCM4 |
| 216237_s_at | -2.8 | -2.1 | -2.6 | -2.6 | -3 | MCM5 |
| 212023_s_at | -3.2 | -2.8 | -13 | -2.5 | -4 | MKI67 |
| 205235_s_at | -2.6 | -2.5 | -4 | -2.6 | -3 | MPHOSPH1 |
| 223381_at | -2.5 | -3.5 | -4.3 | -2.8 | -5.7 | NUF2R |
| 202240_at | -3.2 | -2.5 | -4.3 | -2.3 | -2.6 | PLK |
| 204835_at | -2.8 | -2.1 | -4 | -2.6 | -2 | POLA |
| 205909_at | -3.7 | -2 | -3.5 | -4.9 | -3.5 | POLE2 |
| 218009_s_at | -2.1 | -2.5 | -2.5 | -2.1 | -2.5 | PRC1 |
| 232278_s_at | -2.6 | -3.5 | -3 | -2.3 | -5.7 | SDP35 |
| 223556_at | -2.6 | -2.1 | -2 | -3.2 | -2.3 | SMARCA6 |
| 204886_at | -3.5 | -2.6 | -2.3 | -2.6 | -4.9 | STK18 |
| 218308_at | -2.6 | -2.6 | -6.1 | -2.6 | -4 | TACC3 |
| 223274_at | -2 | -2.6 | -2.6 | -2.1 | -3 | TCF19 (SC1) |
| 201291_s_at | -2.6 | -4.6 | -3.7 | -2 | -5.3 | TOP2A |
| 204822_at | -2.6 | -3.7 | -3.7 | -2.1 | -3 | TTK |
| 204026_s_at | -2.8 | -2.6 | -4.3 | -2.3 | -3.5 | ZWINT |

**Supplementary Table legend**. Cell cycle related genes downregulated in common in K562-PMA, HUVEC-PMA, HUVEC-simvastatin, HUVEC-CRP and HUVEC-atorvastatin
